# Ingestion of A Common Plant’s Leaves Leads to Acute Respiratory Arrest and Paralysis: A Case Report

**DOI:** 10.5811/cpcem.2020.5.46703

**Published:** 2020-07-09

**Authors:** Breelan M. Kear, Richard W. Lee, Sanford B. Church, Fady A. Youssef, Anthony Arguija

**Affiliations:** * University of California, Irvine Medical Center, Department of Emergency Medicine, Orange, California; † University of California, Irvine Medical Center, Department of Internal Medicine, Orange, California; ‡ Memorial Care, Long Beach Medical Center, Department of Pulmonary and Critical Care Medicine, Long Beach, California; § University of California, Irvine Medical Center, Department of Pulmonary and Critical Care Medicine, Orange, California; ** Memorial Care, Long Beach Medical Center, Department of Emergency Medicine, Long Beach, California

**Keywords:** Nicotiana glauca, nicotinic alkaloids, respiratory arrest, toxicity

## Abstract

**Background:**

*Nicotiana glauca* is a plant known to cause acute toxicity upon ingestion or dermal exposure due to the nicotinic alkaloid, anabasine. Nicotinic alkaloids cause toxicity by acting as agonists on nicotinic-type acetylcholine receptors (nAChRs). Initial stimulation of these receptors leads to symptoms such as tachycardia, miosis, and tremors. The effects of high doses of nicotinic alkaloids are biphasic, and eventual persistent depolarization of nAChRs at the neuromuscular junction occurs. This causes apnea, paralysis, and cardiovascular collapse.

**Case Report:**

In this report, we present a case of respiratory arrest due to nicotinic alkaloid poisoning from the ingestion of *Nicotiana glauca*. The diagnosis was suspected after the patient’s family gave a history of the patient ingesting a plant prior to arrival. They were able to also provide a physical sample of the plant.

**Conclusion:**

The phone application, “Plant Snap”, determined the plant species and helped confirm the diagnosis. This case describes how modern technology and thorough history taking can combine to provide the best possible patient care.

## INTRODUCTION

There are several classes of nicotinic and nicotinic-like alkaloid containing plants that can cause toxicological effects in humans.^1^ The plant species in the genus Nicotiana contain these alkaloids. Nicotinic alkaloids exert their toxicity by acting as agonists on nicotinic-type acetylcholine receptors (nAChRs).^1^ Examples of these alkaloids include nicotine, nornicotine, anabasine, and anatabine. In this case report we describe a case of acute toxicity due to the ingestion of the plant, *Nicotiana glauca*, which primarily contains the nicotinic alkaloid, anabasine.^2^

## CASE REPORT

A 65-year-old female was brought to the emergency department (ED) by emergency medical services (EMS) in respiratory arrest. Per EMS report, the patient had felt nauseous and vomited shortly after lunch. One hour later, she was found sitting in a chair complaining of weakness and then suddenly became unresponsive. Her family began cardiopulmonary resuscitation with chest compressions and called 911. Upon arrival to the ED, she was receiving bag-valve-mask ventilation by EMS. She was found to be apneic with a strong pulse. Her initial vital signs were a temperature of 97.5° Fahrenheit, blood pressure of 163/82 millimeters of mercury, heart rate of 61 beats per minute, and 100% oxygen saturation. Her Glasgow Coma Score (GCS) was three, and she had sluggishly reactive mydriatic pupils. She had no signs of trauma. Exam was otherwise unremarkable. She was given intravenous naloxone without a response. The patient was immediately intubated.

The initial workup in the ED included laboratory studies and a computed tomography (CT) of the head. As this was being executed, the patient’s family arrived with further history. Reportedly, the patient suffered from bipolar disorder; however, she had no history of suicide attempts and had been acting behaviorally normal prior to this episode. She was not prescribed any medications, but she did take homeopathic supplements and would occasionally pick dandelions found in her neighborhood for consumption. That morning, she had brought home a new plant “with yellow flowers” and boiled the leaves to eat them. This additional history put a toxicological cause of respiratory arrest higher on the differential; however, other etiologies had not been ruled out.

Laboratory results showed a normal pH on venous blood gas of 7.38, (reference range [Ref]: pH 7.35–7.45); normal electrolytes without anion gap; hyperglycemia of 305 milligrams per deciliter (mg/dL) (Ref: 65–99 mg/dL); mild leukocytosis of 12.5 thousand per microliter (k/μL) (Ref: 3.85k/μL–10.85k/μL); and mild anemia of 10.6 grams per deciliter (g/dL) (Ref: 13.2–17.1 g/dL). The urine drug screen was negative for amphetamines, barbiturates, benzodiazepines, cocaine, methadone, methamphetamines, opiates, phencyclidine, and cannabinoids. Lithium, salicylate, digoxin, cyanide, and acetaminophen were not detected in the serum. The electrocardiogram showed atrial fibrillation at 63 beats per minute with a normal axis. Chest radiograph and computed tomography (CT) of her head were normal.

Given that most of the findings were unremarkable at this point, a “code stroke” was called for fear of basilar cerebrovascular accident. The consulting neurologist noted that the patient had absent oculocephalic and gag reflexes. She had no dystonia or ankle clonus. CT brain perfusion and CT angiography of the head and neck were obtained and were negative. After alternative causes of coma were ruled out, there was increasing suspicion that the plant ingested earlier in the day could be the cause. The patient was admitted to the intensive care unit (ICU).

**CPC-EM Capsule**

What do we already know about this clinical entity?

The response to nicotinic alkaloids found in several plants is biphasic, with initial stimulation of nicotinic-type acetylcholine receptors causing tachycardia, miosis and tremors, and eventual persistent depolarization causing apnea and paralysis.

What makes this presentation of disease reportable?

This case discusses a severe toxic ingestion of a nicotinic alkaloid, anabasine, that led to apnea and paralysis. The phone app,“Plant Snap”, helped to confirm the diagnosis through artificial intelligence and machine learning.

What is the major learning point?

The gold standard of diagnosis for nicotinic alkaloid poisoning are gas chromatography-mass spectrometry and high performance liquid chromatography, both of which were not readily available at the hospital. An easily accessible phone application helped clinch the diagnosis in this case.

How might this improve emergency medicine practice?

Phone applications and new technology can continue to assist us in clinical emergency medicine, but we must continue to perform detailed histories and physicals that lead us toward the correct diagnosis.

**Image 1:**
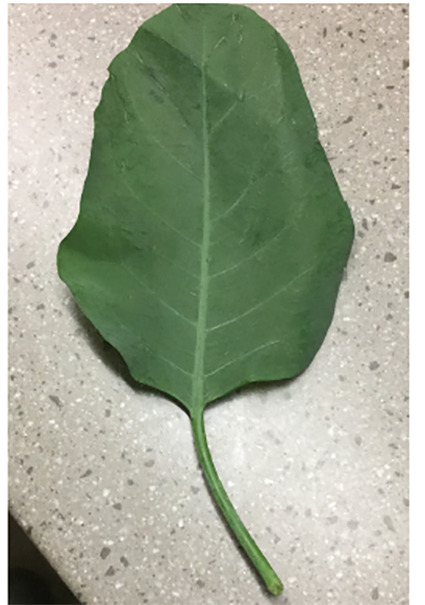
Leaf brought in by the patient’s family, later identified as *Nicotiana glauca*.

Later that evening, the family brought in the plant ingested by the patient to the ICU. A cell phone app, “Plant Snap,” was used to identify the plant as *Nicotiana glauca*. A picture of the plant ingested was sent to Poison Control, which confirmed the identity of the plant (Image 1). Poison Control recommended continuing supportive care. The inpatient team did not pursue confirmatory diagnosis by gas chromatography-mass spectrometry, as it was not readily available at the hospital.

She was initially sedated on a midazolam drip, but this was discontinued after only two hours. She was then started on 25–50 microgram per hour of fentanyl for pain control without any other form of sedation. The next morning, she opened her eyes and seemed to attempt to follow commands, but only exhibited fasciculations of her neck and forearms without true movement. She had an absent gag reflex per respiratory therapist evaluation. Later that afternoon, she began to regain her motor function by weakly moving her extremities. By evening, her gag reflex had returned and she was able to lift her legs off the bed. On day three, she was taking spontaneous breaths on the ventilator, scribbling down words to communicate and demonstrating four out of five upper and lower extremity motor strength. She was extubated on day three of mechanical ventilation and was discharged from the hospital the following day with full recovery. She explained that she had been fully cognizant of her surroundings, but was unable to move during the first two days of admission. She also confirmed that she had ingested several *Nicotiana glauca* leaves after microwaving them.

## DISCUSSION

**Image 2:**
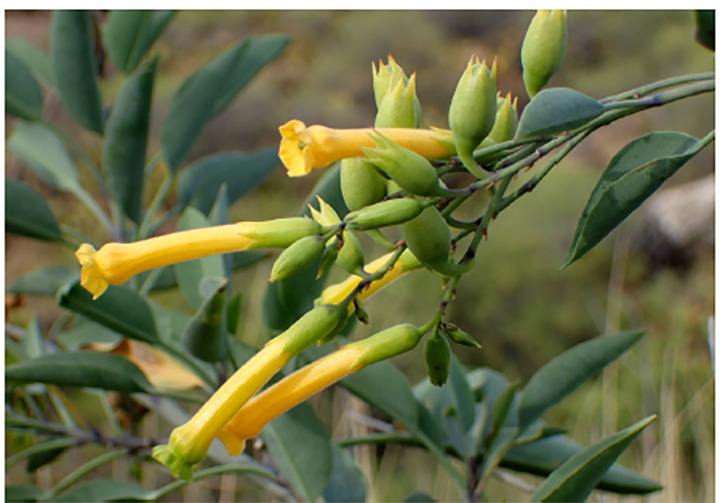
*Nicotiana glauca* including the flowers. Source: Kryzysztof Ziarnek, Kenraiz. File:Nicotiana glauca kz3.JPG. Wikimedia Commons. https://commons.wikimedia.org/w/index.php?curid=47830471. Published March 28, 2016. Accessed May 2, 2020.

*Nicotiana glauca*, or tree tobacco, is a perennial shrub that is native to South America, particularly Bolivia and Argentina. It is now also found in the southwest United States, mainly in southern California, Arizona, and parts of Nevada. Its leaves are long and elliptical shaped, and its flowers are yellow and tubular (Image 2).^3^

*Nicotiana glauca* contains the nicotinic alkaloid, anabasine. It is structurally similar to nicotine, but appears to be more potent.^2^ This similarity allows anabasine to bind to nAChRs, which are located throughout the body, including the neuromuscular junction, as well as the central and autonomic nervous systems.^1^ Anabasine primarily exerts its toxicity by acting as an agonist on these receptors, and the effects are significantly dose related.^1^,^2^ Initially, anabasine binds directly to the nAChRs throughout the body, causing stimulation of both central and autonomic nervous systems.^1^,^4^ Initial symptoms include tachycardia, vomiting, hypertension, and miosis. However, there is a threshold for which even higher doses of anabasine can lead to persistent depolarization of nAChRs at the neuromuscular junction.^1^ This causes a biphasic effect of initial stimulation followed by persistent depolarization, leading to eventual skeletal muscle paralysis, central nervous system collapse, and coma.^1^,^4^,^5^

Symptoms from nicotine and nicotinic-like alkaloid toxicity usually develop within 15–90 minutes of ingestion.^1^,^2^ These symptoms are broad as nAChRs are located throughout the body. Nicotinic alkaloid poisoning classically presents with exam finding of both sympathetic and parasympathetic stimulation due to their activity at both pre- and post-ganglionic nAChRs. Early-phase symptoms include nausea, vomiting, abdominal pain, hypertension, tachycardia, miosis, dizziness, tremors, seizures, and muscle fasciculations.^1^ Delayed phase of toxicity typically occurs within 90 minutes of ingestion and includes respiratory depression, apnea, bradycardia, dysrhythmias, shock, mydriasis, coma, hypotonia, and muscle paralysis.^1^,^2^,^5^ Symptoms can last anywhere from one to two hours in mild toxicity to 24–72 hours in more severe toxicity.^1^,^5^ The patient in the case report experienced the early symptoms of nausea and vomiting after ingesting *Nicotiana glauca* leaves. She rapidly progressed to display the delayed symptoms, ultimately developing complete paralysis, apnea, dysrhythmia, and coma.

Diagnosis of nicotine alkaloid toxicity is typically clinical. In this case, a phone application, “Plant Snap,” was used to identify the specific plant ingested. This application has a database with information on over 600,000 plants, and contains around 250 million images of plants. The user simply takes a picture of the plant, and the application identifies the plant using machine learning and artificial intelligence.^6^ The gold standards of diagnosis of *Nicotiana glauca* poisonings are diagnostic assays using gas chromatography, gas chromatography-mass spectrometry, and high performance liquid chromatography.^7^,^8^

As demonstrated in this case report, the treatment for *Nicotiana glauca* poisoning is largely supportive. Securing the airway with endotracheal intubation followed by mechanical ventilation is paramount in poisonings that cause complete muscle paralysis and thereby respiratory failure. Additionally, seizure control and blood pressure support may be indicated. There is no role for activated charcoal in severe nicotinic alkaloid poisonings where the airway is compromised, but can be considered in mild poisonings that present within one hour. As to prognosis, patients are expected to make a full recovery if effective supportive care is initiated early in the patient’s toxicity.^1^

## CONCLUSION

The differential diagnoses of this case were extremely broad. The initial management of this case included securing the airway as the patient was GCS 3 and apneic. The family’s story about the plant ingestion was not initially presented to the ED team as the patient arrived with EMS. A broad workup was initiated, including studies looking for other toxicological, neurological, and cardiorespiratory causes. Once the common conditionss such as cerebral hemorrhage, basilar cerebrovascular accident, opioid overdose, and hypoxic brain injury were ruled out, it became clear only from further history that this was a case of *Nicotiana glauca* poisoning. Without the additional information from the family, the cause of this patient’s respiratory arrest would not have been known.

The phone application, “Plant Snap,” was paramount in helping us identify the plant as *Nicotiana glauca*. While the plant could likely have been identified by a botanist or using high-performance liquid chromatography, these methods of diagnosis are often not available in a reasonable timeframe. This phone application was free to download and results were available within seconds. There is a growing presence of artificial intelligence across the globe, and this case is an example demonstrating how technology was an aid in our diagnostic acumen in medicine. Nevertheless, in the age of advanced imaging, testing and technology, this case still highlighted the importance of history and physical, while keeping toxicological ingestion high on the differential.
